# Basic Prion Science “Spreads” Insight

**DOI:** 10.1371/journal.ppat.1005092

**Published:** 2015-07-30

**Authors:** David Westaway

**Affiliations:** Centre for Prions and Protein Folding Diseases, University of Alberta, Edmonton, Alberta, Canada

Translational research used to be fun. In fact, it used to be arresting—of course, considered here in the frame of triplets and ribonucleic acids of the transfer and mRNA variety. In fact, the mind-boggling concept that someone could actually *know* the velocity of a ribosome trundling down a messenger RNA, like a freight car on a railway track, led me to decide at a tender age that biochemistry was the only way to go.

On a journey that included a compressed but vigorous stint in Switzerland (“molecular biology boot camp”) and postdoctoral training with an eminent retrovirologist, my budding scientific life stood at a crossroads. What to do next? At this point, I had a reconnection with a moment from the past, in which a second-year, undergraduate version of myself read about an infectious agent called scrapie that didn't have a nucleic acid genome—bizarre! A serendipitous meeting, and this loose end was closed off; several productive research years were spent studying prions in San Francisco.

Fast-forwarding through the nineties and noughties, we come to today’s world, where the N in GANTT doesn’t mean an ambiguous nucleotide but might mean that a progress report needs filing. For most basic biomedical scientists, the drone of the Translational Research mantra to leverage and commercialize can no longer be ignored; that ideology is enacted. The perception that taxpayers are being given value for money is the key driver in this equation, but how best to travel from a research lab environment to a Point A of a deep insight and thence, perhaps, to Point B of a significant impact upon delivered health care or a Point C of a commercialized platform technology?

Perhaps the crux of the dilemma arises from a confusion of expectations and disciplines by politicians. Research and development (R&D) has predictability, a linear trajectory, and short timelines but may offer only shallow insights. R&D is certainly the logical option if one assumes that everything that can be discovered has already been discovered. On the other hand, basic research assumes that the catalog of ground rules for phenomena in the natural world is still being written; it has risk, as it lacks predictability and linearity. A benefit of basic research is that it routinely supplies a rigorous training environment. A yet bigger benefit—albeit less frequent—is that it provides “transformative” insights that can satisfy all expectations and deliverables. However, basic science can suffer from a variation on the theme of giving tax-payers value for money; namely, if the lay public or a Congressman (or, in Canada, a Member of Parliament) can’t understand the project, then they are not satisfied, period—the initiative is deemed wasteful/weird/esoteric and a negative message is directed to the controllers of the research agency.

Prion research has had a profound and lasting impact upon Biology-with-a-capital-B and illustrates these principles with its own trajectory—a trajectory that probably would not have been funded in a purely translational world. The molecular biology of these diseases emerged from the study of an arcane sheep infection that was more agricultural nuisance than life threatening, and with a more dangerous cousin (mad cow disease, Bovine Spongiform Encephalopathy) only coming onto the scene later. Nonetheless, the profound insight that protein shapes can template true-breeding biological information led to a revolution, and this once-heretical idea was helped on its way by being able to solve the mysteries of cytoplasmic inheritance in yeast and fungi. Important parts of the prion story concerning environmental reservoirs and prion-sensitized rodents have been published in the pages of this very journal, with new developments still coming. Ten years ago, the claim that prion fundamentals might apply to common neurodegenerative diseases would have been viewed as delusional, but this idea is now firmly in the mainstream. Diseases like Alzheimer’s and Parkinson’s, with predictable patterns of neuroanatomical progression, behave like this because of an internal infectious process that spreads templating particles within the brain. Furthermore, most of our current animal models of inherited neurodegenerative disease are based on genetic engineering tricks from the prion biology toolbox. Would we have reached this level of understanding without bench research?

The debate about pursuing “doable/shallow” versus “deep/risky” research will undoubtedly go on. However, to my mind, a respected colleague on a grant review panel captured something of the picture for the former with this statement: “That’s not research, that’s just measuring!”

So, what do the taxpayers really want basic research scientists to do?

Measuring tape, anyone?

**Image 1:**
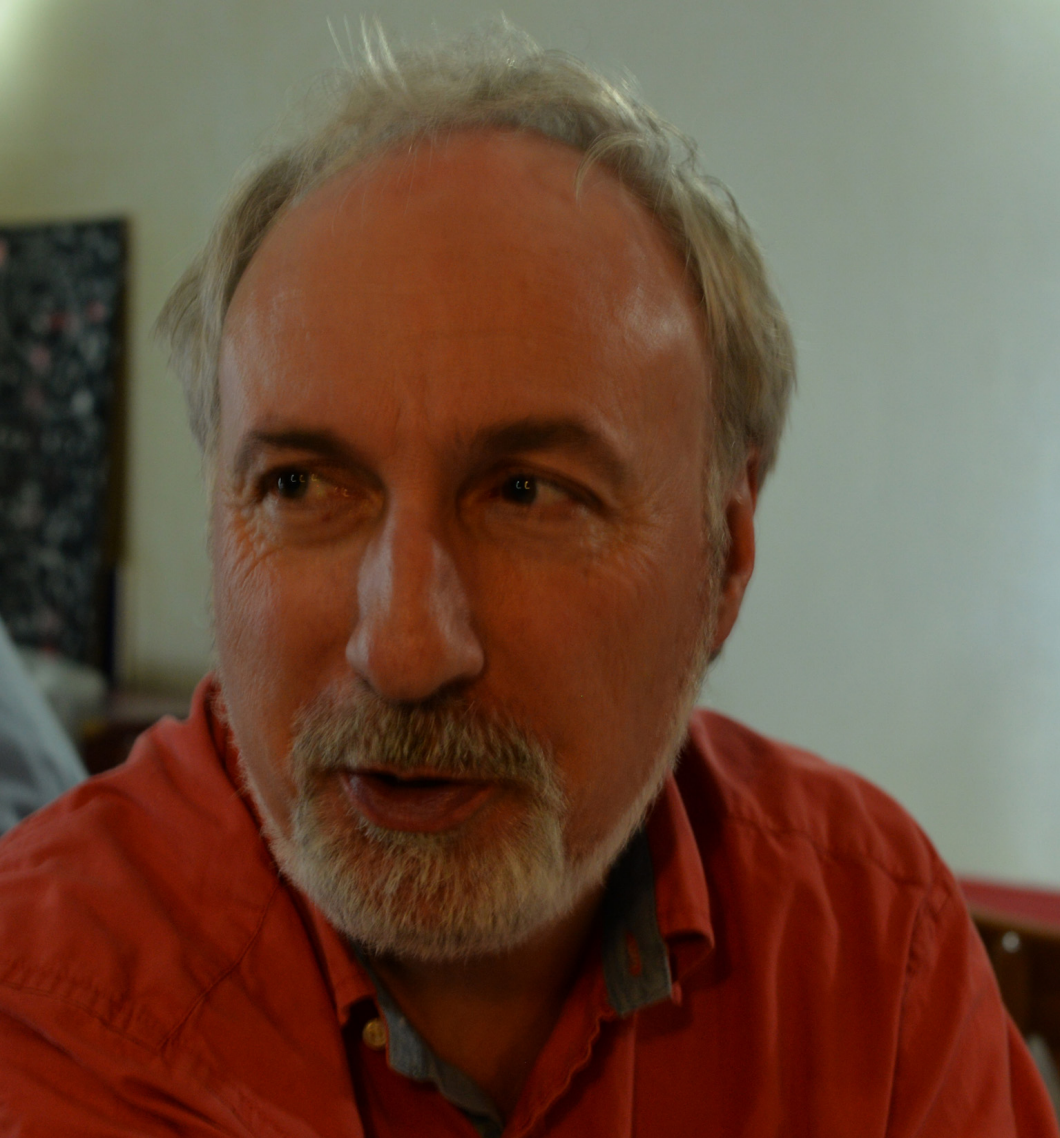
David Westaway.

